# Telemedicine Acceptability Among Patients of Primary Health Care Clinics in the Western Region, Saudi Arabia

**DOI:** 10.7759/cureus.40857

**Published:** 2023-06-23

**Authors:** Saif A Alzahrani, Jumana H Khouja, Saad A GhamdI, Moteab Alotaybi, Amina Bargawi, Abdullmajeed A Alghamdi, Amer Fayraq

**Affiliations:** 1 Preventive Medicine, King Abdullah International Medical Research Center, Ministry of National Guard Health Affairs, Jeddah, SAU; 2 Preventive Medicine, Saudi Board of Preventive Medicine, Jeddah, SAU; 3 Primary Healthcare, King Saud bin Abdulaziz University for Health Sciences, Jeddah, SAU; 4 Preventive Medicine, King Saud bin Abdulaziz University for Health Sciences, Jeddah, SAU; 5 Preventive Medicine, Medical Directorate, Saudi Royal Land Forces, Riyadh, SAU; 6 Preventive Medicine, King Abdulaziz Medical City Jeddah, Jeddah, SAU

**Keywords:** telehealth, sutaq, satisfaction, perceived benefits, healthcare utilization

## Abstract

Background

Telemedicine has become increasingly important during recent years. Investigating the acceptability of telemedicine among patients is an important first step in adapting and maintaining the use of telemedicine and gaining the advantages of technologies in daily practice.

Objective

To measure the acceptability of telemedicine among the patients of primary health care centers (PHCC) using the Service User Technology Acceptability Questionnaire (SUTAQ) at King Abdulaziz Medical City (KAMC), Jeddah, Saudi Arabia.

Methods

This cross-sectional study utilized a validated questionnaire in phone call interviews with patients of PHCC clinics. All patients who had a telemedicine visit at PHCC within the past month of data collection were eligible for inclusion. The SUTAQ tool was used to measure the acceptability of telemedicine technology.

Results

Out of 365 people selected for participation, 73.9% responded. The study found that the median age was 40 years old with an interquartile range of 30-52. The majority of participants were female (61.1%) and married (86.7%). The median total SUTAQ score was 4.3, out of a maximum score of 6. The medians for SUTAQ subscales were as follows: the perceived benefits score was 5.4, the privacy and discomfort score was 2.1, the health care personnel concerns score was 3, the satisfaction score was 5.7, and the kit as substitution score was 4.3. Patients who had not previously experienced telemedicine visits showed a higher score in “health care personnel concerns” (P-value=0.009), while first-time patients had a higher score in “kit as substitution” (P-value=0.006).

Conclusion

This study provided positive evidence that telemedicine is an acceptable service among PHCC patients. However, PHCC providers should prioritize patient education and awareness about telemedicine to improve utilization. Addressing privacy, discomfort, and personnel concerns could increase patient satisfaction. Future studies investigating telemedicine utilization can help in understanding its impact on clinical outcome.

## Introduction

Throughout history, healthcare has benefited from technological advancements, such as imaging techniques, monitoring devices, and robotic surgeries. Telemedicine is another technology that has been increasingly used to deliver healthcare services in recent decades. Telemedicine is defined by the World Health Organization (WHO) as "the delivery of healthcare services, where distance is a critical factor by all healthcare professionals using information and communication technology for the exchange of valid information for the diagnosis, treatment, and prevention of disease, research, and evaluation" [1]. Telemedicine offers benefits such as increased access to healthcare services, reduced travel time and cost for patients, and remote consultation of lab results [2]. The use of telemedicine has become even more crucial during the coronavirus disease 2019 (COVID-19) pandemic due to the unique circumstances of lockdown and limitation of gathering [3]. Positive effects of telemedicine have been reported during the pandemic, such as reducing emergency visits, efficient use of resources, and limiting the spread of infection [4]. Research has shown that synchronous telemedicine visits have high levels of satisfaction among both patients and physicians [5]. In fact, the majority of patients (94-99%) reported being "very satisfied," and one-third even preferred telemedicine over traditional visits [6]. While good acceptance rates were reported, utilization of telemedicine was not always correlated with acceptance [7].

Locally in Saudi Arabia, to provide accessible, safe, and cost-effective care during the pandemic, digital healthcare services have been established [8]. The most commonly used platform for telemedicine in Saudi Arabia was WhatsApp, followed by Zoom and the Seha app developed by the Ministry of Health during the pandemic [9]. Patients during the pandemic reported a high level of satisfaction with the ease, ability to talk freely, and quality of visual technology [10].

Various valid and reliable questionnaires are available to assess different dimensions of telemedicine, such as usability, acceptance, quality, and compliance. However, most of the available tools focus on one to two dimensions either from the patient or healthcare provider perspective [11]. The Service User Technology Acceptability Questionnaire (SUTAQ) was developed through a study conducted in the United Kingdom in 2017 [12] to measure healthcare users' beliefs about the acceptability of a telehealth (TH) service. The SUTAQ questionnaire consists of 22 items, which were measured on a Likert scale from 1 to 6, reflecting varying degrees of agreement with each statement. The questionnaire is composed of five sub-scales, each containing between three and nine items. These sub-scales are improved care, increased accessibility, privacy and discomfort, care staff concerns, alternative toolkit, and satisfaction [12].

Telemedicine has gained popularity in Saudi Arabia's healthcare system in recent times, especially in primary healthcare centers (PHCCs), where access to healthcare services can be limited. However, the acceptability of telemedicine among patients is an important factor that determines its success. Investigating the acceptability of telemedicine among patients is an important first step in adapting and maintaining the use of telemedicine and gaining the advantages of technologies in daily practice. The objective of this study is to measure the acceptability of telemedicine among the patients of PHCCs using the SUTAQ at King Abdulaziz Medical City (KAMC), Jeddah, Saudi Arabia.

## Materials and methods

Study setting

The study was conducted at the PHCC of KAMC in Jeddah, Saudi Arabia. KAMC has 10 PHCCs, but only two centers, Specialized Polyclinic and Bahra, utilized telemedicine and were included in the study. The present study has followed the STROBE (Strengthening the Reporting of Observational Studies in Epidemiology) guidelines to ensure the comprehensive and accurate reporting of our observational research.

In primary care settings, patients have the option to schedule virtual appointments for either new health complaints or follow-up visits related to pre-existing conditions. Additionally, physicians may also assign appointments for follow-up visits as needed. Telemedicine visits for primary healthcare are conducted through a mobile application with an assistant regulating appointments and placing patients in a virtual waiting room. However, patients may not be able to remain in the waiting room for various reasons related to internet connection. In such situations, a phone call is considered more convenient for delivering healthcare through telemedicine clinics. Therefore, both approaches; virtual contact and phone call was considered as telemedicine clinics and their patients are included in our study. 

Study population

The study population consisted of all patients who had a telemedicine visit at PHCC within the past month of data collection, regardless of gender, aged 18 years or older. The one-month duration was chosen to obtain a sufficient number of patients for sampling while minimizing recall bias. Patients who refused to participate were excluded.

With a reference population of 7,000 patients visiting PHCC clinics monthly, the study used an equation with a 5% margin of error, 95% confidence level (CI), and 50% response rate. Using an online software tool (raosoft.com) for calculating sample size for cross-sectional studies, a sample size of 365 was calculated [13]. Out of 365 randomly selected patients, 270 patients responded with a response rate of 73.9%.

A sampling frame was created using data from the electronic system (BestCare) including all eligible patients who met the criteria. BestCare is a Korean-made hospital IT system that was introduced to KAMC in 2014. This hospital information service is an automated system that controls the overall management of the hospital and PHCCs. 

Proportionate sampling was applied to distribute the sample size across the two PHCCs according to the number of patients. Participants were selected using a random number generator in Excel (Microsoft, Redmond, WA, USA).

Data collection

The data collation tool contained three sections. The first section included questions about sociodemographic characteristics. The second section involved questions about the type, time, and duration of the telemedicine visit. The third section was about SUTAQ, which is a validated tool used to measure the acceptability of telemedicine technology [12].

The original English version of SUTAQ was translated to Arabic using the following protocol: forward translation to Arabic by a native Arabic speaker fluent in English, followed by backward translation to English by a native English speaker fluent in Arabic. The final English version was compared and matched with the original SUTAQ form.

The SUTAQ scale's reliability was assessed using Cronbach's Alpha coefficient for the subscales and their respective items. The perceived benefits subscale had a reliability coefficient of 0.86, privacy and comfort subscale had 0.69, health care personnel subscale had 0.70, satisfaction subscale had 0.64, and the kit as a substitution subscale had the lowest reliability coefficient at 0.13. The entire SUTAQ scale had a reliability coefficient of 0.80, indicating high internal consistency. These results show that the SUTAQ scale is a reliable tool for measuring patient satisfaction with telemedicine services.

The study utilized phone call interviews with patients of PHCC clinics. Phone calls were conducted by health care personnel who have been trained on the administration of SUTAQ tool. They started the phone call by introducing themselves and explaining the study nature and purpose to obtain the participants consent. Data sorting and coding started at the end of the data collection period and the completion of the sample size. Data collection took place from April 23 to May 10, 2023.

Data analysis

The statistical analyses were performed using IBM SPSS Statistics for Mac, Version 29.0 (IBM Corp, Armonk, NY, USA) [14]. We assessed the normality of the distribution for continuous variables using the Shapiro-Wilk test. The results of the normal distribution tests were significant (P-value<0.001), indicating skewed distributions of the continuous variables. Median and interquartile range (IQR) were used to summarize the continuous variables. Categorical variables were summarized using frequency tables and proportions. The items of the SUTAQ tool were summed and divided to create a continuous outcome variable ranging from 0 to 6 for each sub-scale. The independent variables were previous telemedicine visits and the reason for the appointment. We used the Mann-Whitney and Kruskal-Wallis tests to investigate the statistical differences between groups in terms of the SUTAQ items. Statistical significance was considered for P-values less than 0.05. No adjustments were made for confounding variables after investigating the demographic characteristics for significant associations with SUTAQ sub-scales.

Ethical considerations

Ethical approval was obtained from the Institutional Review Board (IRB) of King Abdullah International Medical Research Center (KAIMRC) prior to data collection. The approval number assigned was (IRB\0291/23), identified by the study number (NRJ22J/337/12). The interviews began with an explanation of the study's objectives to obtain verbal informed consent from the participants. The participants' contact information was kept with the principal investigator to assure confidentiality. After data collection, phone numbers were removed and replaced by serial numbers before proceeding to data analysis. All data was collected anonymously, without personal identifiers, and used solely for research purposes. 

## Results

The total collected sample was 270 with a response rate of 73.9%. The median age was 40 (IQR: 30-52) years old. Females represented about two-thirds (61.1%) of the total, and the majority (86.7%) of participants were married. In terms of educational level, high school was the most common level attained with 36.30%, followed by less than a high school degree at 27%. A majority (61.50%) of participants reported being unemployed, as the majority of the participants were housewives. The sociodemographic characteristics of the respondents are shown in Table 1. 

The items in the SUTAQ tool were analyzed to calculate the median and IQR for all respondents. The median total SUTAQ score was 4.3 (IQR: 3.7-4.8) out of a maximum score of 6. This total score was further analyzed with respect to the sociodemographic characteristics of telemedicine visitors. The results showed statistically significant variation in technology acceptance among different educational levels. Individuals with higher educational levels had the highest scores, while those with a diploma had the lowest scores for technology acceptance using the SUTAQ tool (P-value=0.043). Other sociodemographic characteristics did not show statistical significance. See the detailed results in (Table 1). 

**Table 1:** Sociodemographic characteristics of the telemedicine beneficiaries SUTAQ: Service User Technology Acceptability Questionnaire

N=270		N (%)	Total SUTAQ (mean rank)	P-value
Gender	Female	165 (61.1%)	128.9	0.272
	Male	105 (38.9%)	139.7	
Marital status	Married	234 (86.7%)	136.6	0.563
	Single	36 (13.3%)	128.5	
Educational level	Less than high school	73 (27%)	121	0.042
	Highschool	98 (36.3%)	142.9	
	Diploma	22 (8.1%)	102.1	
	Bachelor	69 (25.6%)	149.1	
	Higher education	8 (3%)	152	
Employment status	Employed	104 (38.5%)	141.6	0.321
	Unemployed	166 (61.5%)	131.7	

More than half of the appointments (53.7%) were for follow-up visits, followed by investigations results review at 26.7% and new complaints at 14.4%, while other reasons accounted for 5.2% of appointments. There were no significant differences among the sociodemographic characteristics in relation to reasons for telemedicine visits (P-value>0.05). Among males and females, the percentages of appointments for new complaints were 15.2% and 13.9%, respectively. For follow-up visits, the percentage was 50.5% for males and 55.8% for females. The percentage of appointments for investigations results review was the same for both males and females at 26.7%. Among marital status groups, the percentage of appointments for new complaints was 30.6% among single patients, while the lowest was among married patients at 12%. Employed and unemployed respondents both had higher percentages for follow-up visits with 51.0% and 55.4%, respectively. See the details for the reason to visit according to sociodemographic characteristics in Table 2.

**Table 2:** Reasons for telemedicine clinic visit by the sociodemographic characteristics of the patients

	Reason for telemedicine appointment*				P-value
	New complaint	Follow-up	Investigations results	Other	
Gender					
Male	15.2%	50.5%	26.7%	7.6%	0.499
Female	13.9%	55.8%	26.7%	3.6%	
Marital status					
Single	30.6%	50%	16.7%	2.8%	0.022
Married	12%	54.3%	28.2%	5.6%	
Educational level					
Secondary or less	12.3%	56.2%	27.4%	4.1%	0.148
Highschool	13.3%	61.2%	20.4%	5.1%	
Diploma	31.8%	40.9%	27.3%	0.0%	
Bachelor	13.0%	42.0%	36.2%	8.7%	
Higher education	12.5%	75.0%	12.5%	0.0%	
Employment status					
Employed	16.3%	51.%	27.9%	4.8%	0.849
Unemployed	13.3%	55.4%	25.9%	5.4%	

Subsequent analyses of SUTAQ components were calculated. Satisfaction had the highest median score of 5.7 (IQR: 5-6), followed by perceived benefits with a median of 5.4 (IQR: 4.9-5.9). See the details of SUTAQ subscales in Table 3.

**Table 3:** Summary of SUTAQ component scores among primary health care telemedicine patients. SUTAQ: Service User Technology Acceptability Questionnaire

SUTAQ Component	Median Score	Interquartile Range (IQR)
Perceived benefits	5.4	4.9-5.9
Privacy and Discomfort	2.1	1-3.5
Health care personnel concerns	3	2-4.3
Satisfaction	5.7	5-6
Kit as substitution	4.3	3.7-5
Overall	4.3	3.7-4.8

Of the total, 79.6% reported that their recent appointment was their first experience with telemedicine, while 20.4% had previously undergone telemedicine visits. Further analyses were conducted to assess significant differences in technology acceptance among first-time telemedicine visitors and different reasons for visits. Patients who had not previously had telemedicine visits had a significantly higher score in the “health care personnel concerns” item (P-value=0.009). First-time visitors also had a significantly higher score in the “kit as substitution” item (P-value=0.006). Further analyses did not reveal significant differences in item scores between reasons for appointments (Table 4).

**Table 4:** Comparison between the appointment-related factors across the items of SUTAQ tool *P-value calculated using Mann-Whitney test **P-value calculated using Kruskal-Wallis test SUTAQ: Service User Technology Acceptability Questionnaire

	Perceived benefits	Privacy and discomfort	Personnel concerns	Satisfaction	Kit as substitution
	Mean rank				
First appointment					
Yes	149.74	151.6	160.11	129.2	161.09
No	131.86	131.38	129.2	149.83	128.95
P-value*	0.126	0.082	0.009	0.105	0.006
Reason for appointment					
New complaint	131.35	139.45	135.97	123.69	141.32
Follow-up	138.01	138.22	136.51	136.58	133.3
Investigations results	137.47	132.78	132.46	143.52	145.35
Other	110.96	110.32	139.32	115.96	91.36
P-value**	0.633	0.598	0.982	0.408	0.111

Analyses of the study showed that there were significant negative correlations between "perceived benefits" and "privacy and discomfort" as well as "health care personnel concerns" (P-value<0.01). The item "kit as substitution" had significantly positive correlations with all the items (P-value<0.01). Satisfaction was positively correlated with all items except for "privacy and discomfort" and "health care personnel concerns", which showed an insignificant low negative correlation. See the correlation matrix between SUTAQ items in Table 5.

**Table 5:** Correlation between SUTAQ tool items among the patients **P-value <0.01, calculated using Spearman rank correlation coefficient SUTAQ: Service User Technology Acceptability Questionnaire

Correlations	Perceived benefits	Privacy and discomfort	Personnel concerns	Satisfaction
Privacy & discomfort	-.220**			
Health care personnel concerns	-.200**	.657**		
Satisfaction	.585**	-0.076	-0.102	
Kit as substitution	.237**	.316**	.328**	.218**

## Discussion

The current study aims to measure the acceptability of telemedicine among patients of PHCCs using the SUTAQ at KAMC, Jeddah, Saudi Arabia. The study revealed that the median total SUTAQ score was 4.3, indicating a moderate level of technology acceptance among the participants. This finding is consistent with previous research that has consistently shown high levels of patient satisfaction and acceptance of telemedicine, with the overwhelming majority, ranging from 94 to 99%, indicating “highly satisfied” in all aspects of telehealth services. Convenience and decreased travel times and costs are the main drivers of satisfaction [5,6]. During the COVID-19 pandemic in Saudi Arabia, 84.9% of patients who used telemedicine programs found it easy to receive healthcare through telemedicine [10]. The high levels of satisfaction can be attributed to reduced clinic visits and saved travel time. This is evident from the significant reduction in the number of patients who intended to visit emergency departments (ED) after contacting the Sehha application in Saudi Arabia. Sehha is an electronic platform created in 2020 to serve the health sector in Saudi Arabia, providing electronic services approved by the Ministry of Health. This health platform was established in line with the Kingdom's Vision 2030 and in response to government directives. Its aim is to automate, standardize, and facilitate procedures and services in all health authorities. The platform includes many health services under the umbrella of the health system and its various sectors, designed to help individuals from medical facilities. However, the platform name was recently changed to Sehhaty which provides further services [15].

Patients tend to cite the convenience of decreased travel times and costs as the main drivers of satisfaction with telemedicine. There was strong agreement that telemedicine can reduce unnecessary outpatient visits and can be used to monitor chronic patients from home [9]. Our results are consistent with this finding, as reduced travel time was calculated as one of the components within the perceived benefits sub-scale. The overall perceived benefits scored 5.4 out of 6, indicating a high level of importance attributed to these benefits. Moreover, a previous study found that the overall telemedicine acceptance across all demographic groups is effective in reducing the number of hospital visits, with the highest positive statement being increased accessibility factors [16]. The aforementioned perceived benefits of telemedicine, including convenience, improved access to care, and cost savings, were associated with higher levels of technology acceptance, consistent with previous studies that have emphasized the positive impact of perceived benefits on telemedicine acceptance [6,7,17]. In our study, the consideration of telemedicine as a substitute for traditional visits scored 4.3 out of 6, suggesting a moderate level of agreement regarding the use of telemedicine as a replacement. In comparison to previous studies, a majority of patients rated telehealth visits as being just as good as traditional face-to-face visits [5,6]. A study found that most participants' responses ranged between very satisfied and satisfied, especially for ease of registration/scheduling [10].

This study found that the majority of telemedicine appointments were for follow-up visits. This finding suggests that patients are comfortable using telemedicine for ongoing care management, which is in line with previous research demonstrating its effectiveness in reducing unnecessary outpatient visits [16]. Telemedicine provides a convenient alternative to in-person visits, which can help minimize the burden on healthcare facilities and enhance efficiency in healthcare delivery. Moreover, follow-up visits were the most common reason for telemedicine visits across all demographic groups. This is similar to another study where both employed and unemployed respondents had higher percentages of appointments for follow-up visits (51% and 55.4%, respectively) [17]. 

The study revealed negative correlations between the "perceived benefits" component and both the "privacy and discomfort" and "health care personnel concerns" components of the SUTAQ tool. This suggests that patients who believe telemedicine has greater benefits are less worried about privacy and discomfort, and have fewer concerns about healthcare personnel interactions. This finding is consistent with previous research that has emphasized the role of perceived benefits in reducing concerns and increasing acceptance of telemedicine [7,18]. Furthermore, the study found that perceived benefits, such as convenience and improved access to care, were strongly associated with higher levels of technology acceptance. Patients who recognized the advantages of telemedicine were more likely to overcome concerns related to privacy, discomfort, and healthcare personnel issues. These results are consistent with previous studies that have highlighted the positive impact of perceived benefits on telemedicine acceptance [7,17].

This study offers helpful insights for healthcare providers and policymakers who intend to implement telemedicine services in primary healthcare centers in Saudi Arabia. However, there are a few limitations that need to be considered when interpreting the results. The study was conducted in a single center, which may limit the generalizability of the findings to other settings, especially given the cultural differences between different regions. Additionally, the high proportion of female participants (61.1%) and married status (86.70%) may not be representative of the entire population. The study did not investigate the association between factors such as chronic medical conditions and the acceptability of telemedicine, which could provide essential insights for healthcare decision-makers. Finally, it should be noted that while this document reflects the current state of telemedicine technologies, caution should be taken when interpreting the results due to the minor variations in the methods used to conduct telemedicine visits.

## Conclusions

This study provides valuable insights into the sociodemographic characteristics and reasons for appointments of patients utilizing telemedicine services in Saudi Arabia. The findings suggest that follow-up visits are the most common reason for appointments, and patients who had not previously had telemedicine visits had higher concerns regarding healthcare personnel and the use of telemedicine kits as a substitution for in-person visits. Based on these findings, it is recommended that healthcare providers prioritize patient education and awareness regarding telemedicine services to increase patient acceptance and utilization. Additionally, addressing concerns related to privacy, discomfort, and healthcare personnel could further increase patient satisfaction with telemedicine services. Policymakers should prioritize the integration of telemedicine services into the healthcare system and provide adequate training and resources to healthcare providers. However, it is important to consider the potential limitations and challenges of telemedicine for certain populations, such as elderly patients or those with limited access to technology. Healthcare providers should consider these factors when determining the suitability of telemedicine for individual patients and populations. Further studies are needed to explore the long-term effects of telemedicine services on patient outcomes and healthcare utilization in Saudi Arabia.
